# Hypertensive Crisis in Cardiac Tamponade

**DOI:** 10.7759/cureus.2873

**Published:** 2018-06-25

**Authors:** Corina Iorgoveanu, Ahmed Zaghloul, Aakash Desai, Kathir Balakumaran, Kai Chen

**Affiliations:** 1 Internal Medicine, University of Connecticut Health Center, Farmington, USA; 2 Cardiology, University of Connecticut Health Center, Farmington, USA; 3 Cardiology, University of Connecticut Health Center, Farmington , USA

**Keywords:** cardiac tamponade, hypertensive crisis

## Abstract

Cardiac tamponade is a potentially life-threatening disorder believed to be commonly associated with hypotension and even shock. However, many patients with subacute tamponade are indeed hypertensive at initial presentation. We present a case of a 76-year-old female with hypertensive crisis in the settings of cardiac tamponade whose blood pressure normalized after pericardiocentesis.

## Introduction

Cardiac tamponade is caused by the buildup of pericardial fluid (exudate, transudate or blood) that can accumulate for several reasons. The diagnosis of cardiac tamponade is a clinical diagnosis that requires prompt recognition and treatment to prevent cardiovascular collapse and cardiac arrest. The classic physical findings in cardiac tamponade are included in Beck’s triad of hypotension, jugular venous distension, and muffled heart sounds [1].

## Case presentation

**Figure 1:**
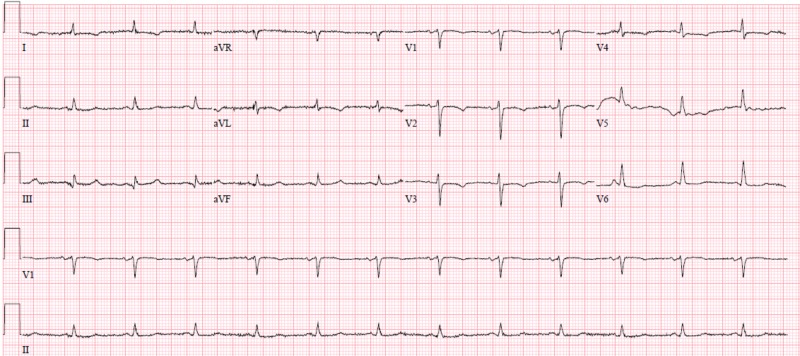
Electrocardiogram on admission. Revealing low voltage QRS and mild electrical alternans with variation of QRS height in consecutive beats.

A 76-year-old female with past medical history of well-controlled hypertension, coronary artery disease presented with subacute progressive shortness of breath for two weeks. On presentation, blood pressure (BP) was 238/146 mm Hg, heart rate (HR) of 75 beats per minute (bpm), SaO_2_ (oxygen saturation) to 80% and was placed on 4 L nasal cannula (NC) with improvement in her oxygenation. Physical exam was remarkable for pulsus paradoxus, distant heart sounds without murmurs or gallops, marked jugular venous distension, diminished breath sounds at the bases and mild bilateral lower extremity pitting edema. Electrocardiogram (EKG) showed only low voltage (Figure 1).

**Figure 2:**
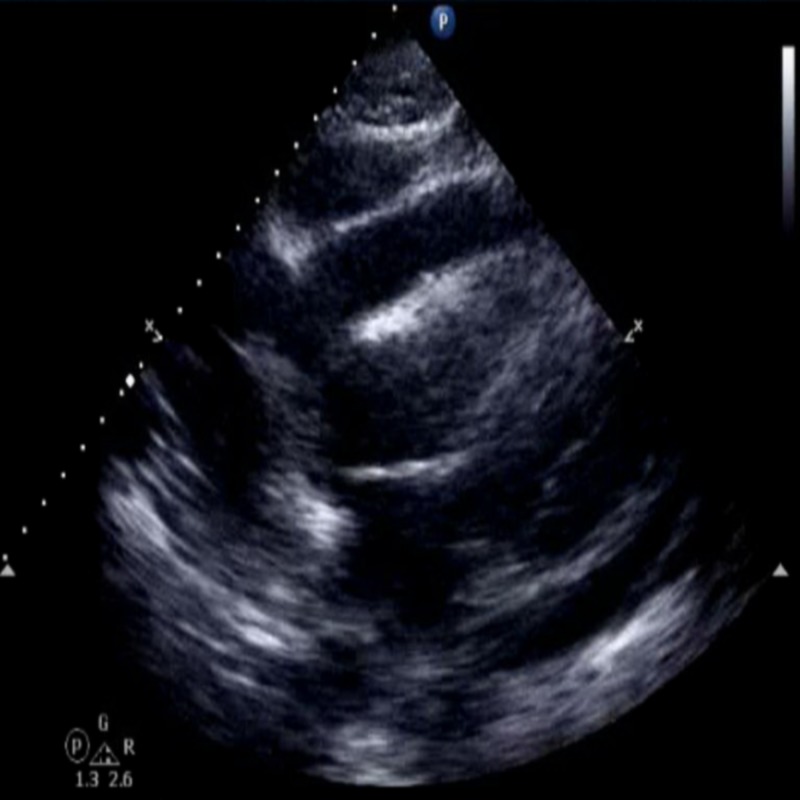
Echocardiogram; subscostal view. Revealing a moderate to large circumferential pericardial effusion surrounding the left ventricular (x).

**Figure 3:**
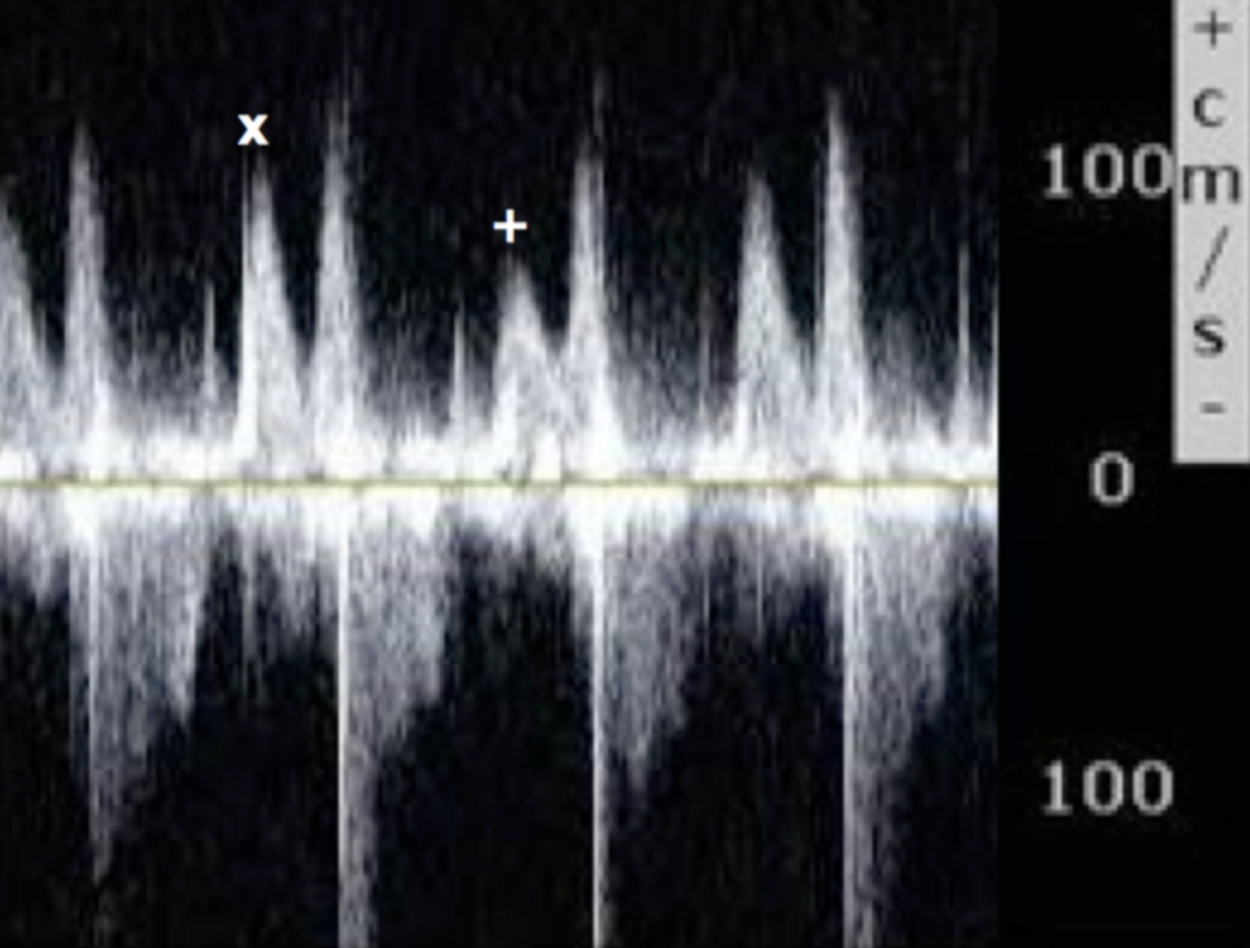
Echocardiogram; pulse wave Doppler across the mitral valve over consecutive beats. Revealing respiratory variation in mitral inflow emptying velocity between expiration (x) and inspiration (+) >25% suggestive of ventricular interdependence.

**Figure 4:**
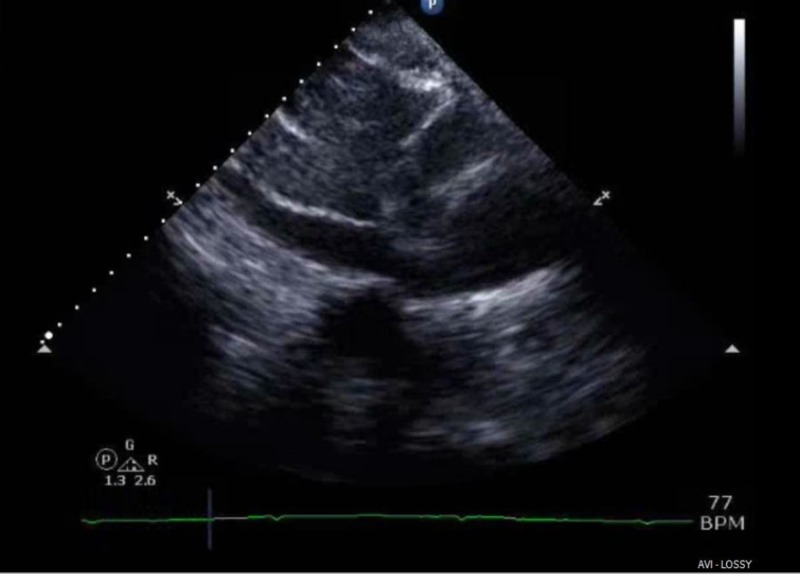
Echocardiogram; subcostal view. Revealing a dilated inferior vena cava (x) >2 cm with blunted respiratory variation indicating elevated central venous pressures.

Chest radiograph showed enlarged cardiac silhouette and bilateral moderate pleural effusions. An echocardiogram demonstrated moderate to large pericardial effusion with tamponade physiology (Figures 2, 3, 4).

The patient remained significantly hypertensive despite adding three antihypertensive medications requiring labetalol drip with failure to control her blood pressure. She underwent pericardiocentesis with the removal of 1200 cc bloody fluid. Right heart catheterization was also done prior and after the pericardiocentesis, which indicated severely elevated right-sided pressures and equalization of right atrial, right ventricular and pulmonary capillary wedge pressure with diminished cardiac output. There was a significant improvement in right-sided pressures following pericardial drainage, with a mean right atrial pressure of 10 mm Hg down from 21 mm Hg. Systemic blood pressure normalized after pericardiocentesis. Follow-up echocardiogram showed resolution of the pericardial effusion. Further workup was done to identify the etiology of the pericardial effusion, including fluid cytology, culture, lactate dehydrogenase (LDH), serum antinuclear antibodies (ANA), serum complement, erythrocyte sedimentation rate (ESR), C‐reactive protein (CRP), anti-double‐stranded DNA (dsDNA), and anti-Smith antibody. Analysis of the pericardial fluid showed exudative fluid and it was negative for malignant cells.

## Discussion

Hypertension with tamponade is an infrequent variant and a number of mechanisms have been described to explain this finding. It is thought that impaired cardiac filling from pericardial fluid accumulation results in a compensatory sympathetic surge that is responsible for increased peripheral vascular resistance and tachycardia [2].

In the patients presenting with tamponade and hypertension, the normalization of blood pressure after pericardial drainage validates the conclusion that the initial elevation in blood pressure was likely a compensatory mechanism due to cardiac tamponade [3].

Brown et al. described a group of six out of 18 consecutive cardiac tamponade patients with hypertension. All of the subjects had a history of chronic hypertension. Following pericardiocentesis, there was a significant decline in peripheral vascular resistance (PVR) in both hypertensive and hypotensive patients. PVR is an important compensatory mechanism, together with increased myocardial contractility, when cardiac output is reduced as a result of pericardial restriction. Pericardiocentesis causes an immediate reduction in intracardiac pressure and improves the cardiac output, thus relaxing the compensatory PVR. Following pericardiocentesis, BPs uniformly decreased in the six hypertensive patients and increased in the hypotensive patients [4].

Argulian et al. directed a retrospective study of eight out of 30 cardiac tamponade patients that presented with hypertension. The findings corroborated with Brown's study and revealed a dramatic decrease in BP in patients who underwent pericardial effusion drainage [5].

It was suggested that β-blockade may counteract hypertensive response in hypertensive tamponade and improve stroke volume by reducing heart rate; however, this idea has not been formally tested. It is also possible that a disturbance of compensatory tachycardia and contractility may prompt decline of cardiac function [4].

## Conclusions

It can be concluded that clinicians should be aware that elevated systemic blood pressure can be present in patients who develop cardiac tamponade. Furthermore, elevated blood pressure resolves after pericardiocentesis. Systemic hypertension should not be used to exclude the diagnosis of cardiac tamponade.

Our case highlights the need to keep cardiac tamponade as a differential in a hypertensive individual presenting with dyspnea.
